# Self-Reported Sleep and Executive Function in Early Primary School Children

**DOI:** 10.3389/fpsyg.2021.793000

**Published:** 2021-12-20

**Authors:** Yulu Chen, Yiji Wang, Si Wang, Ming Zhang, Nan Wu

**Affiliations:** ^1^Department of Psychology, Teachers’ College, Beijing Union University, Beijing, China; ^2^Learning and Psychological Development Institution for Children and Adolescents, Beijing Union University, Beijing, China; ^3^Department of Human Development and Family Studies, Michigan State University, East Lansing, MI, United States; ^4^School of Applied Psychology, Griffith University, Brisbane, QLD, Australia; ^5^Department of Psychiatry, The Third Affiliated Hospital of Sun Yat-sen University, Guangzhou, China

**Keywords:** executive function, inhibitory control, cognitive flexibility, working memory, sleep disturbance, habitual sleep, Sleep Self-Report, cognitive development

## Abstract

The study investigated the associations between children’s self-reported habitual sleep disturbance and multidimensional executive function (EF). Two hundred and four 7–9-year-old typically developing children completed the Sleep Self-Report and finished the Red-Blue Test, Wisconsin Card Sorting Test, and Backward Digit Span Test, indexing different EF components including inhibitory control, cognitive flexibility, and working memory. Results revealed that all the three EF components were significantly correlated with sleep. However, cognitive flexibility was no longer significantly related to sleep when the other EF components – inhibitory control and working memory – were controlled for. Meanwhile, inhibitory control, as well as working memory, was still significantly related to sleep after controlling for the other EF components. Results suggest that children’s self-reported sleep might be associated directly with inhibitory control and working memory, but indirectly with cognitive flexibility.

## Introduction

People spend approximately one-third of their lives asleep. Sleep is essential to healthy development across physical, psychosocial, emotional, and cognitive domains (Sadeh, 2007; Ednick et al., 2009; Dewald et al., 2010; Beebe, 2011; Wang and Yip, 2020). A burgeoning body of research has examined how sleep problems affect cognition (Astill et al., 2012; Shochat et al., 2014; Hoyniak et al., 2019). Theoretically, cognition is a multiple, complex omnibus of psychological abilities (Killgore, 2010), and research has examined the association between sleep and diverse cognitive components including attention, memory, processing speed, and complex reasoning (Lim and Dinges, 2010; Astill et al., 2012). Studies delivered mixed results, with parts of studies revealing the link between sleep and some cognitive abilities, while other studies showing no association in some cognitive tasks (Lim and Dinges, 2010; de Bruin et al., 2017). Such inconsistencies might be attributed to heterogeneous measurement of sleep and cognition, as well as diverse samples from infant to adults, from healthy to atypical populations.

One of the basic and core component of cognitive abilities is executive function (EF). EF refers to a set of fundamental, general mechanisms, and processes, often linked to the prefront cortex of brain, that control, direct, or coordinate cognition and action (Miyake et al., 2000; Lee et al., 2013). EF not only is an essential part for cognition but also works importantly for mental health, school performance, and life success (Alvarez and Emory, 2006; Diamond, 2013). It is often postulated in the literature that EF has three elementary components: inhibitory control, working memory, and cognitive flexibility (also called cognitive shifting; Miyake et al., 2000; Garon et al., 2008; Lee et al., 2013). Each component can be measured by separate tests such as the Stroop test, Wisconsin Card Sorting Test, and digit span backwards test (Miyake et al., 2000; Garon et al., 2008). Research has revealed that the three components were moderately associated with each other but clearly separable (Garon et al., 2008).

As an important domain of cognition, EF has received increasing attention by studies linking sleep to cognition. However, inconsistent findings were reported concerning whether and how sleep was associated with EF (Sadeh et al., 2003; Bourke et al., 2011). After reviewing the relevant literature, we summarized some common aspects as below.

First, most studies examined sleep and multiple types of cognitive abilities including EF (Lim and Dinges, 2010; Astill et al., 2012). In these studies, EF was considered as a sub-domain of the grand cognition, and hence usually only one or two EF tasks were used (Bernier et al., 2013; Sadeh et al., 2015; Vermeulen et al., 2016). Although it is often postulated that there are three different components in EF, few studies have systematically investigated the relationship between sleep and all the components of EF (Karpinski et al., 2008).

Second, studies have often employed experimental manipulation on sleep, such as sleep deprivation/restriction (the most frequently used method; Killgore, 2010; Lim and Dinges, 2010), or bedtime/sleep schedule modification (Sadeh et al., 2003). A meta-analysis of the impact of sleep deprivation on cognitions revealed a moderate effect on working memory (Lim and Dinges, 2010). It is worth noting that such experimental disturbance on sleep was quite acute and drastic, leading to significant impairment of basic arousal and attention and consequently resulting in worse performance on EF tests (Killgore, 2010). Nevertheless, sleep deprivation is not as common as mild or moderate sleep interruption in real-life environment. We therefore can justifiably doubt whether natural and modest disturbance on habitual sleep, such as occasional wake-up at night or daytime sleepiness, has any impact on EF.

Third, another important piece of evidence linking sleep to EF comes from atypically developmental samples. Among these studies, sleep-disordered breathing (SDB), a spectrum of breathing disorders during sleep, from primary snoring to obstructive sleep apnea (OSA) syndrome, was the most frequently assessed sleep index (Beebe et al., 2003; Karpinski et al., 2008; Bourke et al., 2011; Joyce and Dimitriou, 2017). For example, in a study of 3–5-year-old children, the higher parent-reported risk for SDB was associated with impaired EF across the dimensions of inhibitory control, working memory, and planning (Karpinski et al., 2008). In another study of 8–12-year-old children, working memory, a dimension of EF, was found poorer in the OSA group than the control healthy group; poorer working memory was also found associated with sleep-related respiratory index (Lau et al., 2015). Moreover, the findings linking sleep to EF was more likely to be found significant in clinic group such as children with Down syndrome (Joyce and Dimitriou, 2017), mild traumatic brain injury (Landry-Roy et al., 2018), or ADHD (Sciberras et al., 2015). Besides evidence in child samples, a meta-analysis in adult samples also revealed a significant difference in EF between the OSA and control groups (Beebe et al., 2003).

In short of the aforementioned literature, there is evidence showing that drastic sleep disturbance, including sleep deprivation and sleep-disordered breathing syndrome, is linked with worse EF (Beebe et al., 2003; Lim and Dinges, 2010). Yet, scant evidence is available to support the association between mild sleep disturbances and EF in healthy people. Hence, the current study aims to investigate whether individual variation in everyday sleep quality is related to EF using cross-sectional data. In addition, we utilize the multidimensional conceptualization of EF including all the core components of inhibitory control, cognitive flexibility, and working memory (Diamond, 2013). This is what previous research tends to neglect (Karpinski et al., 2008). Thus, the current study will look into all the three EF components and investigate their respective links with habitual sleep disturbance.

Moreover, the previous research on the relation between sleep and EF has primarily examined populations across a broad age range, including samples from children (Astill et al., 2012) to adults (Lim and Dinges, 2010) and even elders (Sforza et al., 2010; Sabeti et al., 2018; Sattari et al., 2019). As previous studies revealed a moderating effect of age (it was found that the relation between sleep and cognition appeared to be more significant in younger people; Bonnet and Rosa, 1987; Sadeh et al., 2002; Dewald et al., 2010), the current study will focus on children rather than adults, seeking to establish the relation between sleep and EF in the early developmental stage.

Furthermore, children’s sleep can be measured by a variety of standardized instruments (Orgilés et al., 2013), including both objective methods (such as polysomnographic technique and actigraphy) and subjective methods (such as self-reported and parent-reported questionnaires). Usually, the objective methods examine the domains of sleep duration and onset time, whereas the subjective surveys can examine more domains, such as bedtime resistance, sleep anxiety, and day-time sleepiness (Owens et al., 2000a). In order to obtain more information on sleep behavior and disturbance, we used questionnaire to measure children’s sleep in the current study. Moreover, given the fact that research shows the self-reported sleep is more closely related to objective assessment than parent-reported sleep (Orgilés et al., 2013; Holzhausen et al., 2021), and that children tend to identify more sleep disturbances by self-report (e.g., sleep onset delay, night waking-up, and daytime drowsiness) than do their parents (Aronen et al., 2000; Owens et al., 2000b), the current study chose a self-reported scale, Sleep Self-Report (SSR; Owens et al., 2000a,b), which has been employed in different countries around the world (van Litsenburg et al., 2010; Schwerdtle et al., 2012; Orgilés et al., 2013; Steur et al., 2019). Considering the fact that the youngest age adapted for SSR is 7 or 8 years old (Owens et al., 2000b; Orgilés et al., 2013), and that the link of sleep and cognition tends to be more significant at younger ages (Dewald et al., 2010), we chose the early primary school children in the current study.

## Materials and Methods

### Participants

Children were mainly recruited from primary schools in the urban area of Beijing, China. Consent from teachers and parents of the participants were obtained before our survey officially started. Participants were all Chinese citizens and native Mandarin speakers, and most of them were from middle-class families. Two hundred and four children (103 boys), aged from 7.6 to 9.2 years (*M* = 8.2 years, *SD* = 0.3 years), were included in the final analysis. Another 28 children were excluded because they had missing or invalid data on either the EF or sleep test.

### Measures

#### Executive Functions

The EF battery was adapted from tests commonly used in developmental psychology. One classic task was chosen for each of the three EF core elements.

##### Inhibitory Control

The Red-Blue Test (RBT) was performed to test children’s inhibitory control. This task was adapted from the classic Stroop (Stroop, 1935) and the Day-Night task (Gerstadt et al., 1994; Simpson and Riggs, 2005). In the RBT task, the children were presented with a red or blue geometric figure on computer screen in each trial. They were told that the red color was named as “blue” while the blue color was named as “red” on an alien planet. The children were required to speak out the color of the figure according to the rule on that alien planet. If a correct answer was given within 2 s, the child would get one score for each trial. There were 36 trials in total. Of note, the original version has 16 trials, which is appropriate for preschoolers. Nevertheless, the current study on early primary school children finally conducted 36 instead of 16 trials, for the following justifications: (1) the previous research (Gerstadt et al., 1994) and our pilot study have found that children tend to make more errors in later trials than earlier trials, and (2) the reliability increases with the number of trials in a cognitive ability test (Steinborn et al., 2018). With the increase of trial number, the current test has gained sufficient reliability (split-half *r* = 0.82, Cronbach *α* = 0.71).

##### Cognitive Flexibility

A simplified version of Wisconsin Card Sorting Test (WCST; Nelson, 1976; Anderson et al., 1991) was conducted on computer to measure children’s cognitive flexibility abilities. In the test of the current study, the children were presented with a screen containing figures with three dimensions (color, shape, and quantity; e.g., two red stars). In each trial, there were one response figure on the top and four stimulus figures on the bottom. The child was asked to match the response figure to one of the stimulus figures according to a particular dimension. Immediately after each trial, feedback on whether the choice was correct or not was given to the respondent. Dimension rule changed every six trials (10 in the original version). When the dimension rule changed, the experimenter would verbally give instruction “Now, let us find out the figure with the same COLOR/SHAPE/QUANTITY of the upper one (target).” (No instruction was specified at the moment in original version). There were 42 trials in total. After the first change of dimension, children would gain one score for each correct trial. Thus, the full mark was 36.

##### Working Memory

The Backward Digit Span Test (Alloway and Alloway, 2010) was performed to test children’s working memory. In the test, the experimenter announced a sequence of numbers two times and the child was required to repeat the numbers in reverse order. The digit length started from two-digit numbers and ended at six-digit numbers. There were three trials for each length. If a child failed in all the three trials of the same length, his/her test terminated. A child would get one score if the correct answer was given within 30 s in each trial. The full score was 15.

#### Sleep

The Sleep Self-Report (SSR; Owens et al., 2000b) was used to measure children’s sleep disturbance in everyday life. The scale was initially designed to assess sleep habits and problems in children aged 7–12 years old. Twenty-three items were asked to rate on a 3-point scale, ranging from “rarely or never (0–1 time per week),” “sometimes (2–4 times per week),” to “usually (5–7 times per week).” Example items include “Do you wake up at night when your parents think you are asleep?,” “Do you feel rested after a night’s sleep? (Reverse scoring).” The scores of these 23 items were then summed up to reflect sleep problem severity: the higher score, the more disturbed sleep.

The scale scores have been demonstrated with high internal consistencies, with a Cronbach’s alpha coefficient of 0.88 in a United States sample (*n* = 334, 6–11 years old; Owens et al., 2000b) and a McDonald’s omega coefficient of 0.85 in a Spanish sample (*n* = 1,228, 8–12 years old; Orgilés et al., 2013).

We developed a Chinese version of the SSR by translating the English source SSR template into Chinese and verifying the translation accuracy through back-translation. Another 433 Chinese children (7–12 years old) were recruited to test the psychometric properties of the Chinese version, revealing that the Cronbach’s *α* was 0.82 and retest-reliability (ICC) was 0.87.

### Procedure

The study was approved by the ethics board of the first author’s university. The children who were enrolled into the current research completed the study in their school. For the three EF tasks, tests were administered on a one-to-one basis between the child participant and a research assistant in a quiet room. The test order of three tasks was counterbalanced. For each of the EF tasks, before formal test, the research assistant introduced the rule and performed two checking trials to ensure the child had fully understood the rule. This session took about 20 min for each child.

For the sleep questionnaire, children were tested collectively in groups of 20–40 children. A research assistant read the instructions and questionnaires aloud (paused about 10 s for each item) and answered any questions arising during the test, so as to ensure that the children had understood and were able to respond to all items. This session took about 15 min.

### Analyses

First, zero-order correlation analysis and partial correlation analysis with the demographic variables controlled for were conducted to examine whether sleep was associated with each of the three EF components (inhibitory control, cognitive flexibility, and working memory). Second, further correlation analyses were employed to investigate the associations between each EF component and sleep after controlling for the other two EF components, respectively. Finally, based on the findings of the second step, path analysis modeling was conducted to examine the complex relationship among sleep and the three EF components.

## Results

### Correlations Between the Sleep and EF

The zero-order correlations between the sleep scores (SSR) and the EF scores (inhibitory control, cognitive flexibility, and working memory) are presented in Table 1. All the three kinds of EF were significantly related to sleep (for inhibitory control, *r* = −0.411, *p* < 0.001; for cognitive flexibility, *r* = −0.325, *p* < 0.001; for working memory, *r* = −0.374, *p* < 0.001), indicating that the more disturbed sleep was associated with worse EF abilities. The partial correlations after controlling for age and sex, as presented in Table 1, showed that the association between sleep and EF also held.

**Table 1:** Descriptive statistics & zero-order/partial correlations among study variables (*N* = 204).

	Sex	Age	Sleep, *SSR*	Inhibitory control	Cognitive flexibility	Working memory
Zero-order (above diagonal) and partial (below diagonal) correlations after controlling for sex and age						
Sex	—	0.025	0.090	−0.042	0.116	−0.072
Age		—	0.095	0.084	0.058	0.109
Sleep, *SSR*			—	−0.411^***^	−0.325^***^	−0.374^***^
Inhibitory control			−0.420^***^^,^ ^a^	—	0.453^***^	0.412^***^
Cognitive flexibility			−0.336^***^^,^ ^a^	0.470^***^^,^ ^a^	—	0.465^***^
Working memory			−0.385^***^^,^ ^a^	0.404^***^^,^ ^a^	0.488^***^^,^ ^a^	—
Partial correlations after controlling for the other two EF components as well as sex and age						
Sleep, *SSR*				−0.272^***^^,^ ^b^	*−0.086* ^c^	−0.213^**^^,^ ^d^
Descriptive statistics						
*M* (*SD*)	0.5 (0.5)	8.2 (0.4)	37.4 (5.5)	33.5 (2.7)	31.8 (3.1)	9.3 (2.6)
*Range*	0–1	7.6–9.2	24–57	26–36	23–36	4–15
Skewness	0.02	0.48	−0.03	−1.28	−0.78	−0.07
Kurtosis	−2.02	0.17	0.37	1.54	0.37	−0.66

*Sex, 1 = girl, 0 = boy; M = Mean. SD = Standard Deviation. SSR = Sleep Self-Report, a 23-item, 3-point scale to measure children’s sleep, where higher score of SSR indicates more sleep disturbance and worse sleep behavior. Italics are partial correlations*.

a *Control variables: sex, age.*

b *Control variables: cognitive flexibility, working memory, sex, age.*

c *Control variables: inhibitory control, working memory, sex, age.*

d *Control variables: inhibitory control, cognitive flexibility, sex, age*.

*** *p < 0.001*;

** *p < 0.01*.

Results also revealed that the three components of executive functions were moderately correlated with each other, suggesting that they were constructed on a common element.

### Correlations Between the Sleep and EF After Controlling for Other EF Components

Given the fact that the different components of EF are associated with each other, sleep might be statistically associated with one EF component (e.g., shifting) in an indirect way *via* taking effect on another one or two EF components (e.g., inhibitory control or working memory) which are naturally related to the former EF component (e.g., shifting).

Based on the justification above, we conducted further partial correlations between sleep and EF controlling for the other two EF subcomponents as well as sex and age (Table 1). For inhibitory control, its correlation with sleep held after adding into cognitive flexibility and working memory as controlled variables (*r* = −0.272, *p* < 0.001). For working memory, after additionally controlling for the inhibitory control and cognitive flexibility, its link with sleep was also significant (*r* = −0.213, *p* = 0.004). For cognitive flexibility, however, there was no significant association with sleep after additionally controlling for the other two EF subcomponents (*r* = −0.086, *p* = 0.227).

### Path Analysis Examining the Complex Relationship Between Sleep and Different Components of EF

**Figure 1:**
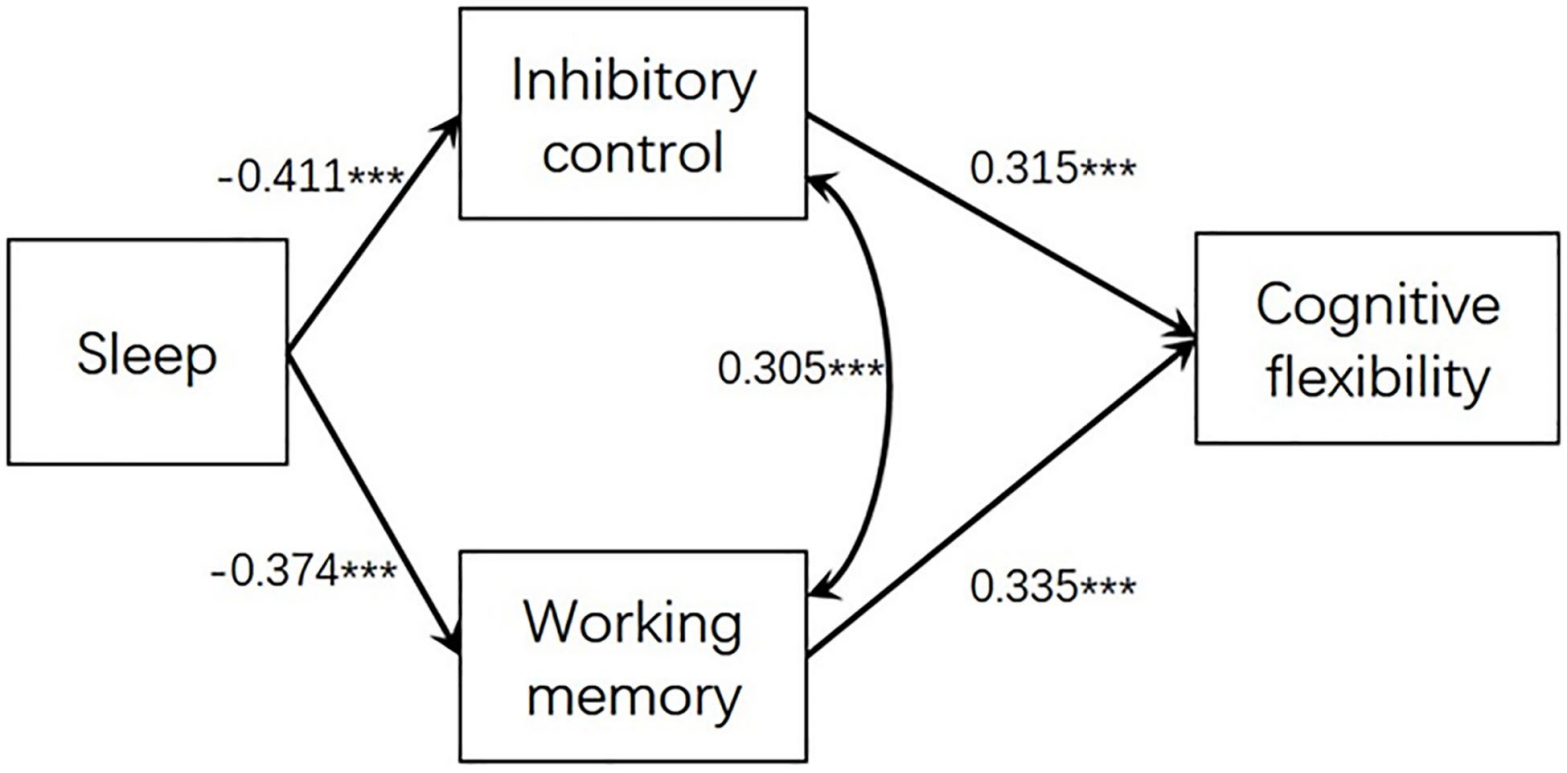
Path analysis modeling examining the indirect association between sleep and cognitive flexibility *via* inhibitory control and working memory; *** *p* < 0.001.

As we found that the association of sleep with cognitive flexibility turned non-significant after controlling for inhibitory control and working memory, we further conducted a path analysis model to investigate the mediation effect of inhibitory control and working memory between sleep and cognitive flexibility. The model was computed by M*plus* 7.0 and the method of robust maximum likelihood estimation was chosen. The impacting paths and coefficients are presented in Figure 1. The model testing showed a good model fit: *χ*^2^(1) = 1.513, *p* = 0.219, CFI = 0.996, TLI = 0.979, SRMR = 0.019, RMSEA = 0.050. The indirect effects from sleep to cognitive flexibility *via* inhibitory control (coefficient = 0.130, *SE* = 0.032, *t* = 4.023, *p* < 0.001) and *via* working memory (coefficient = 0.125, *SE* = 0.035, *t* = 3.607, *p* < 0.001) were both significant.

We also conducted another comparative model by freeing the path parameter from sleep to cognitive flexibility. Results showed that the direct path from sleep to cognitive flexibility was not significant (*b* = 0.090, *SE* = 0.171, *t* = 1.272, *p* = 0.203) and the indirect effects from sleep disturbance to cognitive flexibility *via* inhibitory control and *via* working memory remained significant (*p*s < 0.001).

## Discussion

The current study examined whether and how sleep disturbance in everyday life was related to the different components of executive function in typically developing children. Previous research mainly focused on the effect on EF from sharply disrupted sleep, such as sleep deprivation and chronic sleep breathing disorder (Jones and Harrison, 2001; Beebe et al., 2003; Killgore, 2010; Lim and Dinges, 2010). Among these studies, EF, which actually is a multidimensional concept including three core components of inhibitory control, flexibility, and working memory (Diamond, 2013), was usually regarded as just one of the facets of cognition, and only one or two EF tasks were measured. In our study, the self-reported sleep score focusing on several aspects of habitual sleep, such as inadequacy of sleep, nocturnal disturbance, and day-time sleepiness, was found to be correlated to scores of all the three EF components. We further found that, while the other two EF components – inhibitory control and working memory – were controlled for, cognitive flexibility was no longer significantly related to sleep. Meanwhile, the partial correlation between sleep and inhibitory control after controlling for working memory and cognitive flexibility, as well as the one between sleep and working memory after controlling for inhibitory control and cognitive flexibility, was still significant. Results suggest that self-reported everyday sleep disturbance in children might be directly associated with working memory and inhibitory control, but only indirectly linked to cognitive flexibility.

Previous studies have yielded inconsistent results on whether sleep disturbance is associated with compromised EF in children (Killgore, 2010). After comparing these studies, we found that the different aspects of sleep measured by different methods might account for the discrepancy. Specifically, positive results were mostly provided by studies using questionnaire to measure multiple aspects of sleep, while negative results were mainly from studies using actigraphy or sleep dairy to measure sleep duration or efficiency (the ratio of minutes asleep to minutes in bed). For example, a significant association was found between scores of Sleep Self-Report questionnaire and working memory of 5–13-year-old children (Sciberras et al., 2015), but not found between those of sleep duration (or sleep efficiency) and working memory of 7–13-year-old children (Sadeh et al., 2002). The heterogeneous result patterns of subjective multiple sleep questionnaire and objective sleep duration can also be identified in the same study. For example, in a study of 8–9-year-old children, working memory was found associated with sleep scores from questionnaire (School Sleep Habits Survey) but not to sleep duration from actigraphy (Buckhalt et al., 2007). Similarly in an adolescent study, the correlation between sleep scores computed from Epworth Sleepiness Scale and EF scores was found significant, while sleep duration provided by actigraphy was not significantly associated with EF outcomes (Anderson et al., 2009). It was argued that sleep questionnaire and actigraphy-derived sleep duration might tap into different aspects of sleep (Landry-Roy et al., 2018). In the current study, we used a questionnaire concerning multiple aspects of sleep including habit and disturbance; therefore we could find the association between sleep and EF. Results from the current study and the studies mentioned above suggest that the multiple aspects of sleep, rather than the sleep duration, could be significantly associated with EF development of children. Of note, it does not mean that adequate sleep duration is not important for children’s cognitive development. A meta-analysis showed that the effect of inadequate sleep on simple attention (the more basic cognitive component) was “large,” while its effect on EF including working memory and short-term memory was just “small” (Lim and Dinges, 2010). We therefore speculate that different aspects of sleep are associated with different parts of cognition.

The complex relationship in which sleep disturbance is directly associated with inhibitory control and working memory while indirectly linked with cognitive flexibility might be attributed to the comprehensive inter-structure of EF itself. It is suggested and has been evidenced that the three components of EF share some common bedrock but have their own separate parts (Garon et al., 2008). Each EF component is supposed to have a non-executive common part to which the other two components also share, and an executive specific part which it holds exclusively. Research has proven that sleep deprivation can affect the executive and non-executive processes in different patterns (Tucker et al., 2010). In order to interpret the results in the current study, it may be safe to conclude that habitual sleep disturbance in children may be associated with the common part as well as the specific parts of working memory and inhibitory control but may not be linked to the specific part of flexibility. There was converging evidence yielded in other studies: it was found that the performance on Wisconsin Card Sorting Test, which indexed cognitive flexibility, was not significantly associated with one-night sleep deprivation (Steinhoff and Eckardt, 1999) or consecutive days of partial sleep deprivation (Khazaie et al., 2010). These results suggest that cognitive flexibility might be not sensitive to sleep problems.

The authors were also concerned that the correlation between sleep and EF in the present study might be resulted from some confounding variables. The first considered factor in developmental research was age, so we controlled for children’s age in the subsequent partial correlation analysis, and the results revealed that the association between sleep and EF remained significant. Besides, other possible noise variables (such as socio-economic status and parenting styles) that were not measured in the present study might explain the association between sleep and EF in children. However, our further examination suggested that confounding variables could not account for the whole correlation between sleep and EF. If the common correlated part among sleep and the three EF components could be completely explained by the confounding variables, then all the three correlations between sleep and each of the EF components would completely disappear after controlling for the other two EF components, since that had completely encompassed the common correlated part shared by all the EF components and sleep. However, the analysis of partial correlation between inhibitory control and sleep after controlling for the other EF components, as well as that between working memory and sleep after controlling for the other EF components, denied the case. It suggested there might be, at least, some extra or specific link between sleep and inhibitory control, and between sleep and working memory, even when the common covariate factors were ruled out.

The current study was just a correlational study and could not reveal the direction of the relationship. Hence, the finding of the link between sleep and EF could be resulted from either the effect of sleep on EF or the effect of EF on sleep. Both of the two directions are possible to work in everyday life. Nevertheless, we tended to interpret the current result as that sleep might have an impact on EF development: on one hand, there were numerous studies employing sleep deprivation evidenced that sleep did impact several cognitive abilities including some EF elements (Lim and Dinges, 2010); on the other hand, there was also evidence from neuroscience research, which demonstrated that several important neural processes benefit from sleep. For example, sleep would facilitate the process of synaptic downscaling (or homeostasis), which makes the excessive activity of neurons during the performance of specific information-processing in the day-time into an energetically suitable level (Tononi and Cirelli, 2006; Makela et al., 2020). Sleep at night would also help the upregulation of protein associated with brain and synaptic plasticity (Basheer et al., 2005; Cirelli, 2005). These molecular biological processes benefitted from sleep are the neural basis for the development of cognitive abilities including EF. Further, the particular relationship between sleep disturbance and the development of EF ability can be interpreted through the maturation of the prefrontal region of the brain. Evidence from cognitive neuroscience studies suggested that the development of prefrontal cortex is particularly vulnerable to the effect of sleep disturbance (Harrison et al., 2000). Meanwhile, the EF ability was proved to be strongly associated with prefrontal cortex (Alvarez and Emory, 2006; Garon et al., 2008). Hence, it is speculated that the disruption of sleep leads to subsequent prefrontal cortical dysfunction, resulting in the poor performance in the EF test.

To our best knowledge, the current study is the first to investigate the complex relationship between self-reported sleep disturbance in everyday life and all the three basic EF subcomponents in typically developing children, revealing that the correlations between each of the EF components and sleep were all significant but the positive association between sleep and cognitive flexibility did not hold after controlling for the other two EF (inhibitory control and working memory). These findings advance current knowledge about the relationship between sleep and cognitive development. Yet, there are limitations to be considered in future research. In the current study, all the EF performances were measured by accuracy, not by reaction time (RT). Since some researchers suggested that RT or speed is more reliable in certain cognitive tests (Steinborn et al., 2018), such as in the current inhibitory control test (Lagattuta et al., 2011), more indexes or tasks are needed in further investigation. Moreover, as the current study only tested early primary school children in China, it remains to be evaluated whether the same result pattern will emerge in individuals from other countries, or from other age-stages (such as preschoolers, adolescents, or adults). In the context of cognitive development, some evidence shows that the structure of EF does not remain stable but develops with age. For example, by using factor analysis of different EF tests, the component of shifting was found to be mixed with inhibition when the children are young (Lee et al., 2013). For this reason, we might expect there are possibly different correlation patterns among EF tasks and sleep in different age groups. In addition, as we have discussed above, sleep scales and sleep duration derived from actigraphy might tap into different aspects of sleep that might impact EF differently. Given the fact the current study only used the Sleep Self-Report scale, future research should utilize both objective and subjective methods to measure sleep.

## Data Availability Statement

The raw data supporting the conclusions of this article will be made available by the authors, without undue reservation.

## Ethics Statement

The studies involving human participants were reviewed and approved by the Ethics Committee of Psychology Department, Beijing Union University. Written informed consent to participate in this study was provided by the participants’ legal guardian/next of kin.

## Author Contributions

YC, MZ, and NW conceived the study. YC, YW, and SW analyzed the data. YC and YW drafted the manuscript. MZ and NW revised the manuscript. All authors contributed to the article and approved the submitted version.

## Funding

This research was supported by the Beijing Social Science Foundation (No.16JYC023), Scientific Research Project of Beijing Education Committee, Premium Funding Project for Academic Human Resources Development in Beijing Union University to YC, Science and Technology Planning Project of Guangdong Province (No.2017A070713010) to MZ, Academic Research Projects of Beijing Union University (No.JS10202002) to NW.

## Conflict of Interest

The authors declare that the research was conducted in the absence of any commercial or financial relationships that could be construed as a potential conflict of interest.

## Publisher’s Note

All claims expressed in this article are solely those of the authors and do not necessarily represent those of their affiliated organizations, or those of the publisher, the editors and the reviewers. Any product that may be evaluated in this article, or claim that may be made by its manufacturer, is not guaranteed or endorsed by the publisher.
