# Supplementary material for: Self-Reported Sleep and Executive Function in Early Primary School Children
Source: Front Psychol. 2021 Dec 20;12:793000. doi: 10.3389/fpsyg.2021.793000 (PMC8720750; doi:10.3389/fpsyg.2021.793000)
Supplement: Supplementary file 1 [file Table_1.DOCX]

**Self-reported sleep and executive function in early primary school children**

**Supplementary materials**


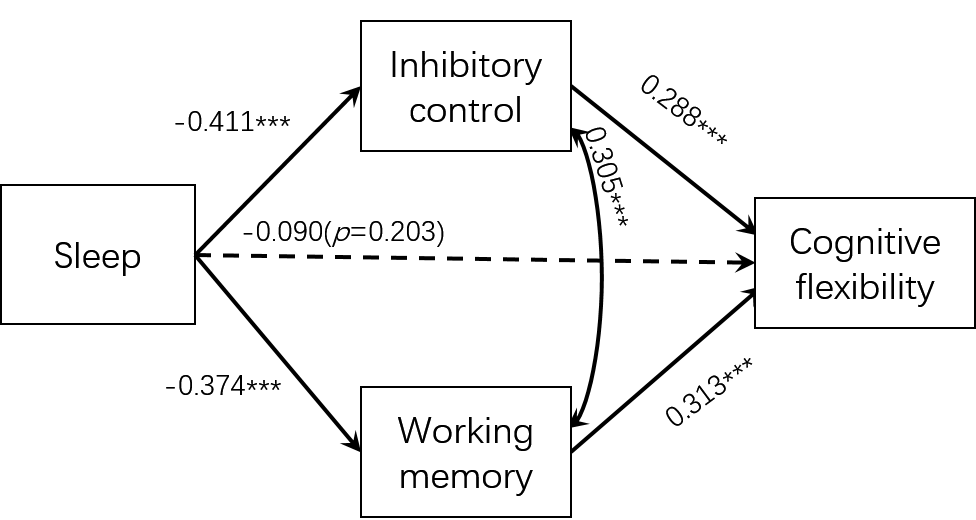


**Figure 1s.** Comparative model with the change of freeing the path parameter from sleep to cognitive flexibility. The coefficients of indirect effects from sleep to cognitive flexibility via working memory and via inhibitory control were 0.117 (*SE* = 0.034, *t*= 3.483, *p* < 0.001) and 0.118 (*SE* = 0.033, *t* = 3.564, *p* < 0.001).

*** *p*<0.001
